# Sentinel lymph node-guided modification of postoperative radiotherapy in oral tongue squamous cell carcinoma: a retrospective dosimetric and NTCP modelling study

**DOI:** 10.1016/j.ctro.2026.101306

**Published:** 2026-10-08

**Authors:** Einar Björgvinsson, Aeneas Kolev, Eva Onjukka, Mattias Hedman, Åsa Carlsson Tedgren, Lars Södergren, Signe Friesland, Gregori Margolin, Lars-Olaf Cardell, Michael Gubanski

**Affiliations:** a Department of Head and Neck Oncology, Karolinska University Hospital, 17164 Stockholm, Sweden; b Department of Oncology-Pathology, Karolinska Institution, 171 77 Stockholm, Sweden; c Department of Head and Neck Surgery, Karolinska University Hospital, 17164 Stockholm, Sweden; d Department of Science, Intervention and Technique, Karolinska Institution, 141 52 Stockholm, Sweden; e Department of Nuclear Medicine, Karolinska University Hospital, 17164 Stockholm, Sweden; f Department of Radiation Therapy, Karolinska University Hospital, 17164 Stockholm, Sweden

**Keywords:** Head and neck cancer, Sentinel lymph node biopsy, Postoperative radiotherapy, Radiotherapy volume optimization, Normal tissue complication probability

## Abstract

• SLNB identified lymphatic drainage beyond standard elective volumes in nearly half of OTSCC patients.
• SLNB may guide individualized postoperative radiotherapy, including omission of contralateral neck irradiation in selected patients.
• Adapted plans significantly reduced estimated NTCPs for xerostomia and dysphagia.
• SLNB may support individualized de-escalation and optimization of elective neck irradiation in OTSCC.

## 1 Introduction

In Sweden, the annual incidence of oral tongue squamous cell carcinoma (OTSCC) is approximately 175 newly diagnosed cases [1]. At Karolinska University Hospital, the internal protocol of treatment for all patients except those with stage I disease consists of primary surgical resection and postoperative radiotherapy (PORT) to the primary site combined with bilateral elective neck irradiation.

In recent years, sentinel lymph node biopsy (SLNB) performed at the time of surgery has been established as the standard approach for cervical lymph node staging. In addition, SLNB is currently used to guide the inclusion of elective lymph node target volumes for PORT by adding neck levels harboring sentinel node (SN) not included in the standard treatment protocol. SLNB has been demonstrated to be reliable, with a high sensitivity and a negative predictive value exceeding 90% for cervical lymph node staging [2], [3]. This reliability is clinically relevant, as regional recurrence accounts for up to 70% of all recurrences and constitutes a major cause of poor prognosis in this patient group [4], [5]. In addition, SLNB can reveal aberrant lymphatic drainage, including drainage to the contralateral neck.

PORT is commonly used for OTSCC with published guidelines for its indications [6], [7]. Due to the sensitive structures in the head and neck region, minimizing irradiated volumes is desirable. Excess morbidity, including dysphagia, oral mucositis, xerostomia, and hypothyroidism, is closely related to doses and volumes [8], [9], [10], [11], [12]. Despite advances in conformal radiotherapy (RT) techniques, irradiation of elective neck levels still involves substantial exposure of normal tissue. Standardized atlases of neck regions for guiding RT are published with description of anatomically projected drainage patterns to different neck levels [13]. However, these do not consider possible individual drainage patterns, which may potentially result in omitting irradiation of levels at risk for metastasis.

It remains unclear whether SLNB results can be applied to modify PORT to improve safety or reduce side effects. We hypothesize that SLNB could identify regions at risk for metastasis, which then can be added to the elective volumes that would otherwise be omitted, thereby increasing safety. Furthermore, absence of contralateral uptake might allow omission of the contralateral neck, substantially reducing irradiated volumes and doses to organs at risk (OAR). To examine this, we retrospectively constructed plans based on the original PORT target volumes and compared them to modified adapted plans using SLNB results as a guide. We also estimated the potential reduction in side effects by omitting the contralateral neck if no contralateral drainage was present. This was assessed using normal tissue complication probability (NTCP) models for selected OAR.

## 2 Methods

This retrospective study includes 42 patients who were previously enrolled in a trial on SLNB in OTSCC, conducted at the Head and Neck Department, Karolinska University Hospital, between April 2016 and December 2021 [14]. All participants underwent clinical examination, tumor biopsy, and computed tomography (CT) imaging, followed by staging according to the Union for International Cancer Control (UICC) 8th edition TNM classification at a multidisciplinary conference. The inclusion criteria were OTSCC of any T and N stage treated with curative intent surgery including SLNB and PORT. The exclusion criteria were prior surgery or RT in the head and neck region and distant metastatic disease. The study was approved by the regional ethics committee in Stockholm (protocol numbers 2013/1943–31/4, 2019–03518 and 2024–01475-02), and all participants provided written informed consent.

### 2.1 Sentinel node biopsy protocol

Static imaging using single-photon emission computed tomography (SPECT-CT) was conducted 1 to 12 h following submucosal injection at four points (one in each quadrant) adjacent to the tumor, utilizing the radiotracer Technetium-99 m (^99^mTc). Thirteen patients received 99mTc-labeled colloidal human serum albumin (Nanocoll®, GE Healthcare), while 29 patients were administered ^99^mTc-labeled tilmanocept (Lymphoseek®, Cardinal Health). Surgical procedures were performed within 24 h post-injection, with dosage adjusted based on the interval to surgery. Intraoperative localization of SN was facilitated using a handheld gamma probe (Europrobe®, Euro-Medical Instruments) to detect radiotracer activity. An ipsilateral selective neck dissection of levels I-III was performed, with additional levels or lymph node biopsies as required based on SN location. The procedure concluded with the resection of the primary tumor in the oral cavity.

### 2.2 Radiotherapy and chemotherapy

All patients received external beam radiotherapy (EBRT) using a Varian© linear accelerator (Palo Alto, CA, USA) and patients receiving brachytherapy were treated with an ^192^Ir afterloader from Elekta© (Stockholm, Sweden), see Table 1. In 8 patients, concomitant chemotherapy with weekly cisplatin was administered at the discretion of the treating physician if adverse features were present in the pathology report.

**Table 1:** Radiotherapy received by the patient cohort.

	Node-negative disease (*n* = 26)	Node-positive disease (*n* = 16)
Tongue dose-prescription schedules	A) 2 Gy × 23–25 (*n* = 21)	A) 1.52 Gy × 34 (*n* = 6)
	+ 18.5–24 Gy BT boost* (*n* = 20)	+ 18.5–24 Gy BT boost* (*n* = 6)
	B) 2 Gy × 34 to resected side and 1.8 Gy × 34 to unresected side (*n* = 4)	B) 2 Gy × 34 to resected side and 1.52 Gy × 34 to unresected side (*n* = 5)
	+ 18.5 Gy BT boost (*n* = 1)	+ 8.4 Gy BT boost* (n = 1)
	C) 2 Gy × 34 to resected side and 1.52 Gy × 34 to unresected side (n = 1)	C) 2 Gy × 25 (*n* = 3)
		+ 24 Gy BT boost* (n = 3)
		D) 2 Gy × 34 to resected side and 1.8 Gy × 34 to unresected side (*n* = 2)
Metastatic node level prescription	–	A) 2 Gy × 34 (*n* = 13)
		B) Elective only (microscopic metastases) (*n* = 3)
Elective nodal prescription	2 Gy × 23 (seq. boost)	1.52 Gy × 34 or 1.86 Gy × 25
	1.52 Gy × 34 (SIB)	
Elective nodal irradiation	Bilat levels I-III (*n* = 19)	Bilat levels I-III + ipsilat level IV (*n* = 16)
	+ bilat levels IV (*n* = 4)	+ Contralat level IV (contralateral involvement)
	Ipsilat levels I-III (*n* = 1)	+ Ipsilateral level V (*n* = 5)
	Contralat levels I-IV (n = 1)	
	None (n = 1)	
External-beam boost technique	Sequential boost or SIB^	SIB^

*High dose-rate or pulsed dose-rate brachytherapy over 4 days

^SIB = simultaneous-integrated boost

BT = brachytherapy

### 2.3 Retrospective treatment planning and dose optimization

While the clinical treatment plans varied in modality and included volumes, the hypothesis was explored by retrospectively simulating alternative plans for each patient, following a uniform EBRT-only treatment strategy. Simultaneous-integrated boost (SIB) plans were generated based on the original target volumes; however, if treated unilaterally, a bilateral target volume was instead delineated as reference. The delineated target volumes for the adapted plans are described below. All plans were optimized in ARIA/Eclipse© version 16.1 and included two full 6 Megavoltage arcs.

To standardize dose optimization and minimize user dependence, knowledge-based auto-planning was applied using the same locally trained RapidPlan© model (Varian, Palo Alto, CA, USA) for both the original and the adapted treatment plans. The RapidPlan model was trained on 111 historical cases of head and neck cancer treatment plans. Optimization criteria at time of study are documented in **Appendix A.**

### 2.4 Creation of adapted radiotherapy plans

SLNB results were documented for each patient, including the presence of lymphatic drainage to neck levels outside the standard treatment protocols and the absence of contralateral drainage. Furthermore, we defined two patient groups: one with normal and one with reduced risk respectively. For each patient, a modified adapted PORT plan was generated based on the SLNB findings and dosimetrically compared with the original plan. If the patients original plan included bilateral nodal irradiation that was used for comparison. For the few patients that had unilateral nodal irradiation or omission of elective volume altogether a plan with bilateral neck irradiation was created for comparison and generalizability with elective dose of 46 or 51.68 Gy to neck levels I-III contralaterally. The procedure is shown in Fig. 1.

**Fig. 1:**
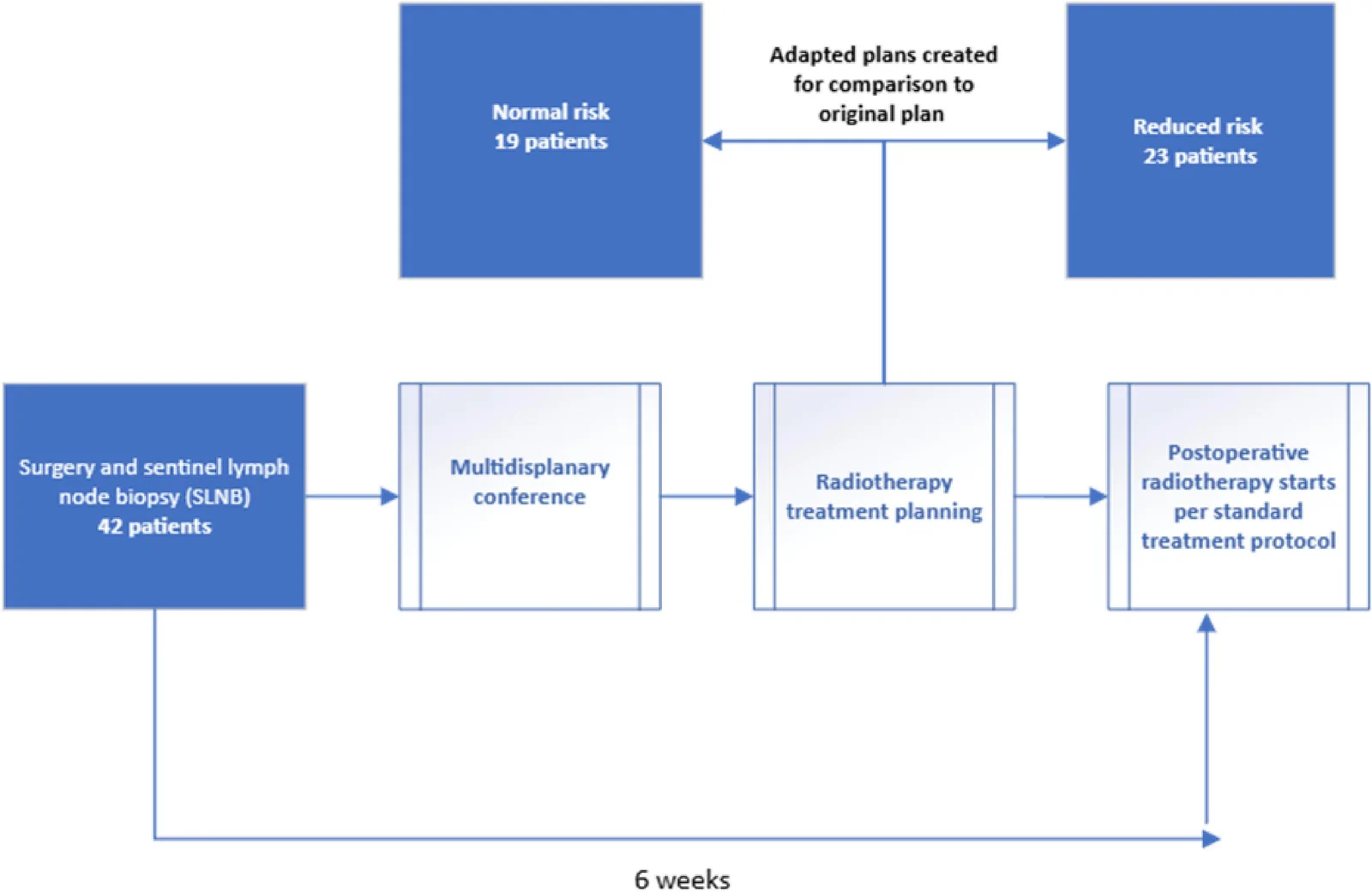
Illustration of workflow creating adapted plans.

#### 2.4.1 Normal risk group

For patients with either pN+ disease or the presence of a contralateral SN an adapted treatment plan was created. If a SN was located at a neck level not covered by the standard treatment protocol, the adapted plan also incorporated the non-standard level as well as any intermediate levels. Example of a patient in this group is visually represented in Fig. 2 **IA**.

**Fig. 2:**
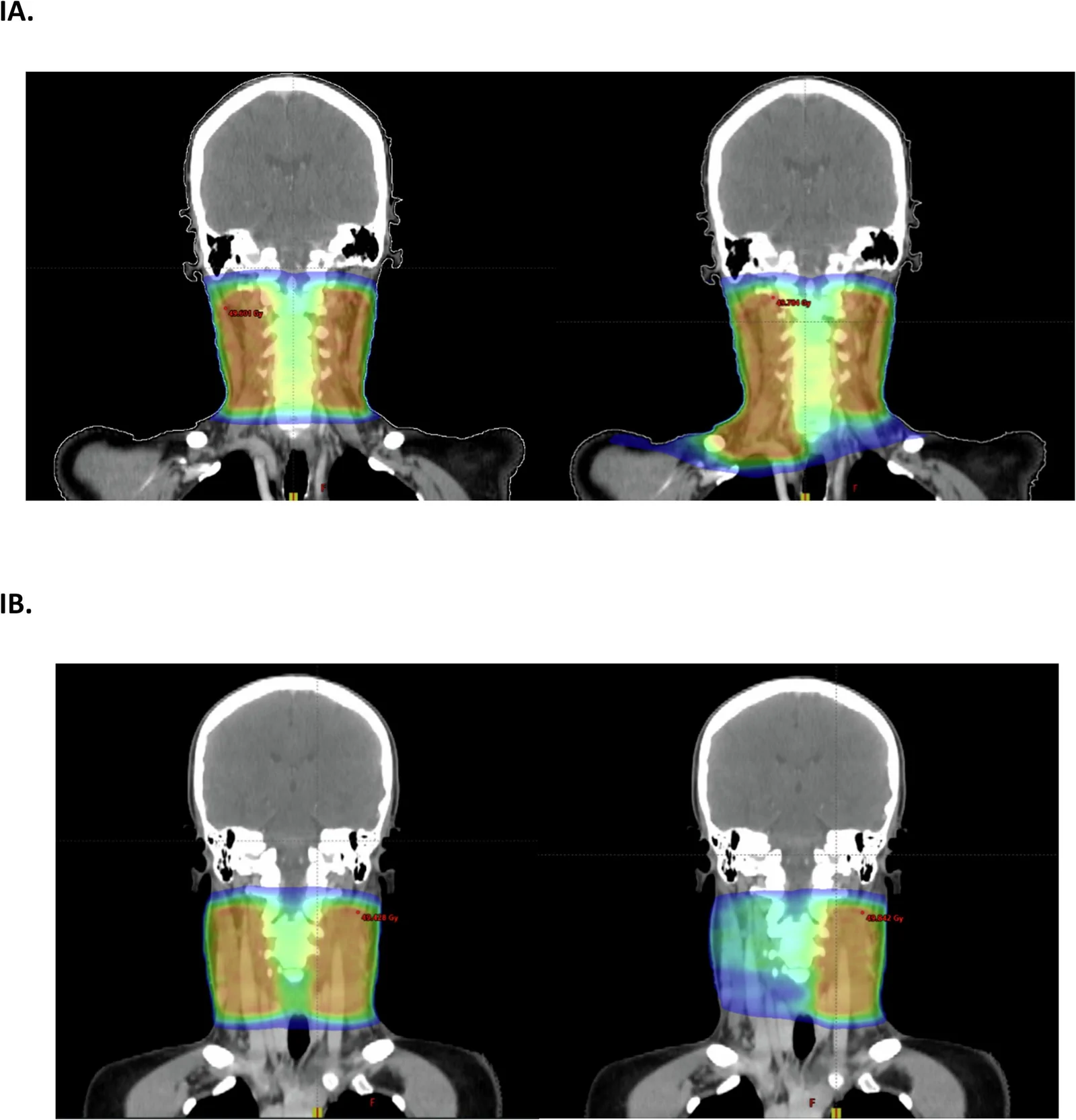
IA Patient in normal risk group illustrating added neck level not in the original plan; IB Patient in reduced risk group illustrating omission of the contralateral neck.

#### 2.4.2 Reduced risk group

For patients with pN0 status and no SN identified contralaterally, an adapted plan was created omitting the standard contralateral volume entirely. If, however, a SN was concurrently present ipsilaterally at a non-standard elective level, this level was added to the plan. One patient with pN+ disease and micrometastatic nodal involvement was also included in this group, given the low estimated risk of contralateral metastasis. An example of a patient in this group is visually represented in Fig. 2 **IB**.

### 2.5 Estimated effect of SN based treatment planning

To estimate the clinical impact of the adaptive SN-based workflow, the SN-adapted plan was compared with the original. If no modification of the treatment volume was required, the two plans were identical. For each case, doses to OARs were compared using NTCP models for three toxicity endpoints: dysphagia, xerostomia and acute oral mucositis. Relevant published models were selected from the literature presented in Table 2 and applied to extracted dose-volume histograms (DVHs) for corresponding OAR. DVHs were first converted to equivalent dose in 2 Gy fractions (EQ.D2) using α/β = 3 Gy.

**Table 2:** Summary of NTCP models used for different toxicity endpoints and references.

Endpoint	Grade 3+ xerostomia	RTOG/EORTC grade 2+ dysphagia	G3+ mucositis
Time after RT	12 months	6 months	8 weeks
OAR	Total parotid volume	Superior pharyngeal constrictor muscle + supraglottic larynx	Oral cavity
DVH parameter	Mean dose	Mean dose	Mean dose
Model type	Lyman-Kutcher-Burman	Multivariable logistic regression	Logistic regression
Reference	Lee et al. 2013 [16]	Christianen et al. 2012 [19]	Bhide et al. 2012 [18]

OAR = Organs at Risk, DVH = Dose-Volume Histogram, ROTG = Radiation Oncology Toxicity Grading, EORTC = European Organization for Research and Treatment of Cancer.

For xerostomia and dysphagia, where several models have been published, model selection was based on principles presented in a publication by Langendijk et al. [15]. Models were favored if they were based on patients treated with modern techniques such as intensity modulated radiotherapy (IMRT) and showed good prospective assessment of patient-rated complications with high compliance rates. The number of patients and events had to be adequate with good model performance (discrimination and calibration) and available original datasets. Furthermore, multivariable analysis with internal validation was preferable. For dysphagia, we used the same model as selected in the Langendijk publication, whereas for xerostomia we applied a different model proposed by Lee et al. [16].This model evaluates the dose to the total parotid volume, rather than only the contralateral parotid as in Beetz et al. [17], allowing for a more complete and robust comparison between the plans. For oral mucositis, few models have been published, which is why we selected the one that fitted best with the previously mentioned criteria [18].

### 2.6 Statistics

NTCP calculations were performed using published model definitions (Table 1). The NTCP values for the original- and SN-adapted plans were compared with Wilcoxon paired sample test in Matlab (Mathworks, Massachusetts). A p value less than 0.05 was deemed as significant.

## 3 Results

In our cohort of 42 patients the median age was 63 years (range 22–78). Demographics, smoking history and stage of disease are summarized in Table 3**.**

**Table 3:** Demographics, smoking history and stage.

Patients *n* = 42	
Mean age at diagnosis	63 (range 22–78)
	N (%)
	
Sex	
Female	16 (38)
Male	26 (62)
Smoking	
Yes	15 (36)
No	27 (64)
Side of tongue	
Left	17 (40)
Right	25 (60)
Clinical stage before surgery	
T1	10 (24)
T2	22 (52)
T3	10 (24)
T4	0
N0	38 (90)
N1	1 (3)
N2b	3 (7)
M	0
Pathological stage after surgery	
T1	7 (17)
T2	19 (45)
T3	16 (38)
T4	0
N0	26 (62)
N1	8 (19)
N2b	4 (10)
N2c	1 (2)
N3b	3 (7)

At diagnosis, 32 patients (76%) had clinical stage T1–T2 tumors, whereas 26 (59%) had the corresponding pathological stage following surgery. Clinically, 38 patients (90%) were staged as N0 prior to surgery, while 26 (62%) were confirmed to have pathological N0 postoperatively (pN0).

### 3.1 SN-status

The distribution of SN is summarized in Table 4. Nineteen patients (45%) had a SN at a level not covered by the time standard treatment protocol. Among these, the majority had SN in the ipsilateral region IV and V. Two patients had SN in the contralateral region IV. Thirty-five patients (83%) had no contralateral SN indicating no lymphatic drainage to the contralateral side. In fifteen patients (36%) positive SN was reported by the pathologist. The most prevalent location of positive SN was in levels II and III, which ipsilaterally occurred in 10 and 6 patients respectively. Level V and Ib ipsilaterally were involved in 2 patients respectively and only one patient had positive SN contralaterally in level II.

**Table 4:** Distribution of sentinel node (SN).

	Patients; N = 42 (%)
No Contralateral SN	35 (83)
SN not included in standard treatment protocol	19 (45)
Neck levels with SN not included in standard treatment protocol:	
IV ipsilaterally	15 (36)
V ipsilaterally	8 (19)
IV contralaterally	2 (5)
	
Positive SN	15 (36)
Levels with positive SN	
II ipsilaterally	10 (24)
III ipsilaterally	6 (14)
V ipsilaterally	2 (5)
Ib ipsilaterally	2 (5)
II contralaterally	1 (2)

### 3.2 Estimated effect of SN based treatment planning

The median doses to OAR and NTCP values are summarized in Table 5. For the reduced risk group, a comparison between the adapted plans and the original plans showed a significant reduction (*p* < 0.01) in the estimated median risk of severe xerostomia (grade 3+) by 2.9 (IQR: 1.4–4.0) percentage points (pp) and moderate to severe late dysphagia (grade 2+) by 2.9 (IQR: 2.5–3.9) pp., respectively **(**Fig. 3**).** For severe acute mucositis (grade 3+) the reduction was 0.2 (IQR: 0.0–0.4) pp.

**Table 5:** Mean doses and NTCP, median (inter-quartile range), for original and adapted plans, respectively, reported both for the reduced risk group and all patients. ΔNTCP (also illustrated in Fig. 2, Fig. 3) shows the median (inter-quartile range) of the pair-wise difference in NTCP, per endpoint. The p value indicates the statistical significance by the Wilcoxon paired sample test.

		Reduced-risk subgroup		All patients	
		Original plans	Adapted plans	Original plans	Adapted plans
Xerostomia	Parotid Dmean (Gy)	13.7 (10.8–17.5)	8.9 (8.1–11.4)	14.2 (11.5–18.4)	11.6 (8.5–14.7)
	NTCP (%)	7.6 (5.8–10.5)	4.9 (4.5–6.2)	7.9 (6.2–11.3)	6.2 (4.7–8.3)
	ΔNTCP (pp)		−2.9 (−4.0 to −1.4)		−0.6 (−2.9–0.0)
	p (Wilcoxon)		<0.001		<0.001
Dysphagia	PCM Dmean (Gy)	41.5 (37.9–43.3)	38.1 (36.4–41.5)	42.9 (39.5–48.9)	37.5 (34.5–40.6)
	SG larynx Dmean (Gy)	35.3 (31.3–38.0)	25.5 (21.7–29.3)	37.5 (34.5–40.6)	33.2 (25.5–39.3)
	NTCP (%)	8.4 (6.2–9.3)	5.1 (3.7–6)	8.9 (8.9–8.9)	6.9 (5.0–12.1)
	ΔNTCP (pp)		−2.9 (−3.9- -2.5)		−1.4 (−3.2–0)
	p (Wilcoxon)		<0.001		<0.001
Mucositis	Oral cavity Dmean (Gy)	40.5 (38.8–42.3)	40.5 (38.3–41.4)	41.5 (39.3–44.8)	41.2 (38.9–44.9)
	NTCP (%)	44.3 (43.2–45.3)	44.3 (42.9–44.8)	44.9 (43.5–46.8)	44.7 (43.2–46.8)
	ΔNTCP (pp)		−0.2 (−0.4–0.0)		0.0 (−0.2–0)
	p (Wilcoxon)		0.001		0.003

PCM = pharyngeal constrictor muscle, SG = supraglottic, pp = percentage points.

**Fig. 3:**
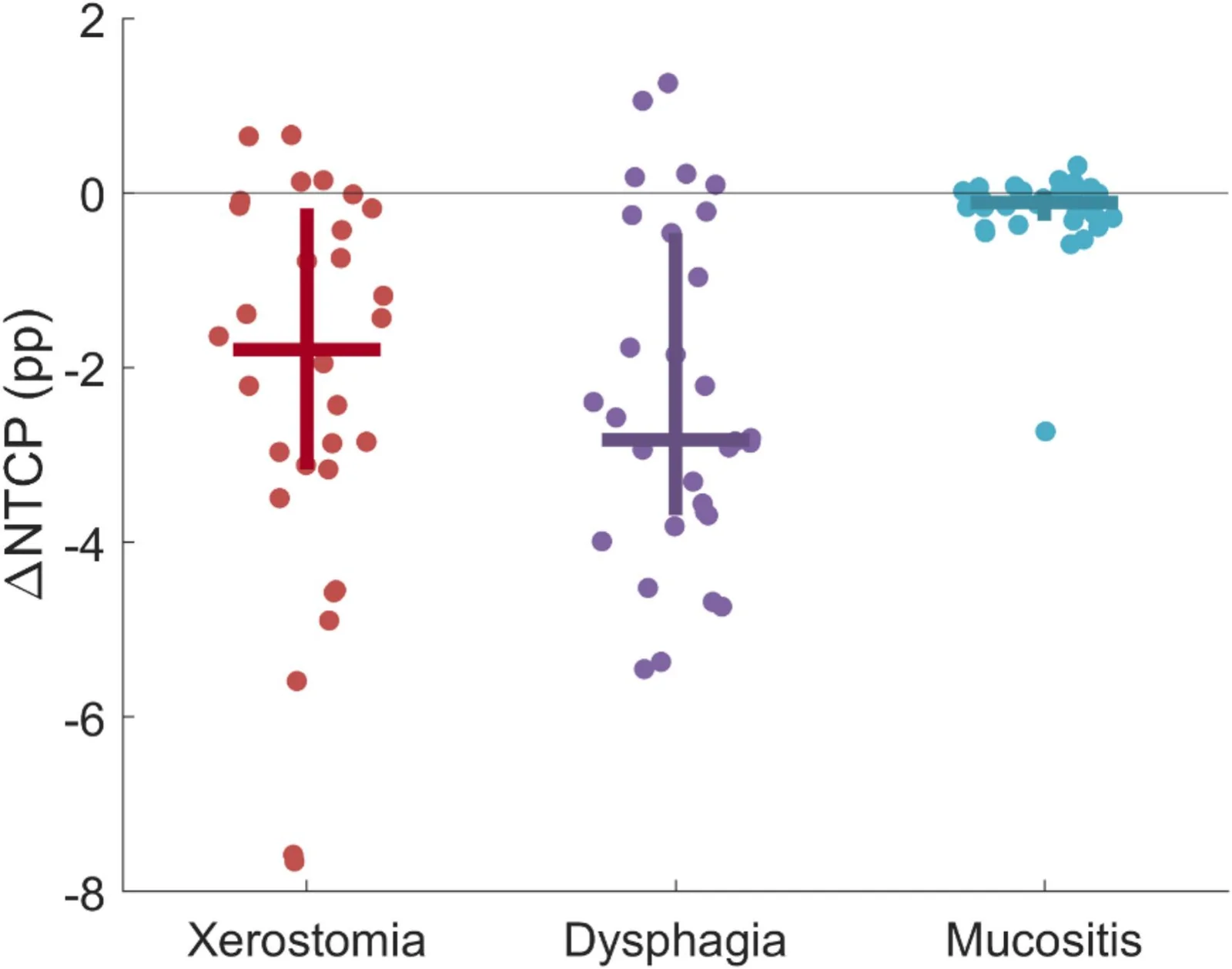
Difference in NTCP between the adapted plans and the original plans in the reduced risk group, in percentage points (pp).

To estimate the effect of the adaptive SN based workflow, the NTCP was compared between the adapted plans and the original plans for the complete cohort (including the patients with normal risk) **(**Table 5**,** Fig. 4**).** Where no adapted plan was required, a difference of zero was recorded in relation to the original plan. It showed a significant reduction (*p* < 0.01) in the estimated median risk of severe xerostomia of 0.6 (0.0–2.9) pp. of moderate late dysphagia of 1.4 (0.0–3.2) pp. and of sever acute mucositis of 0.0 (0.0–0.2) pp.

**Fig. 4:**
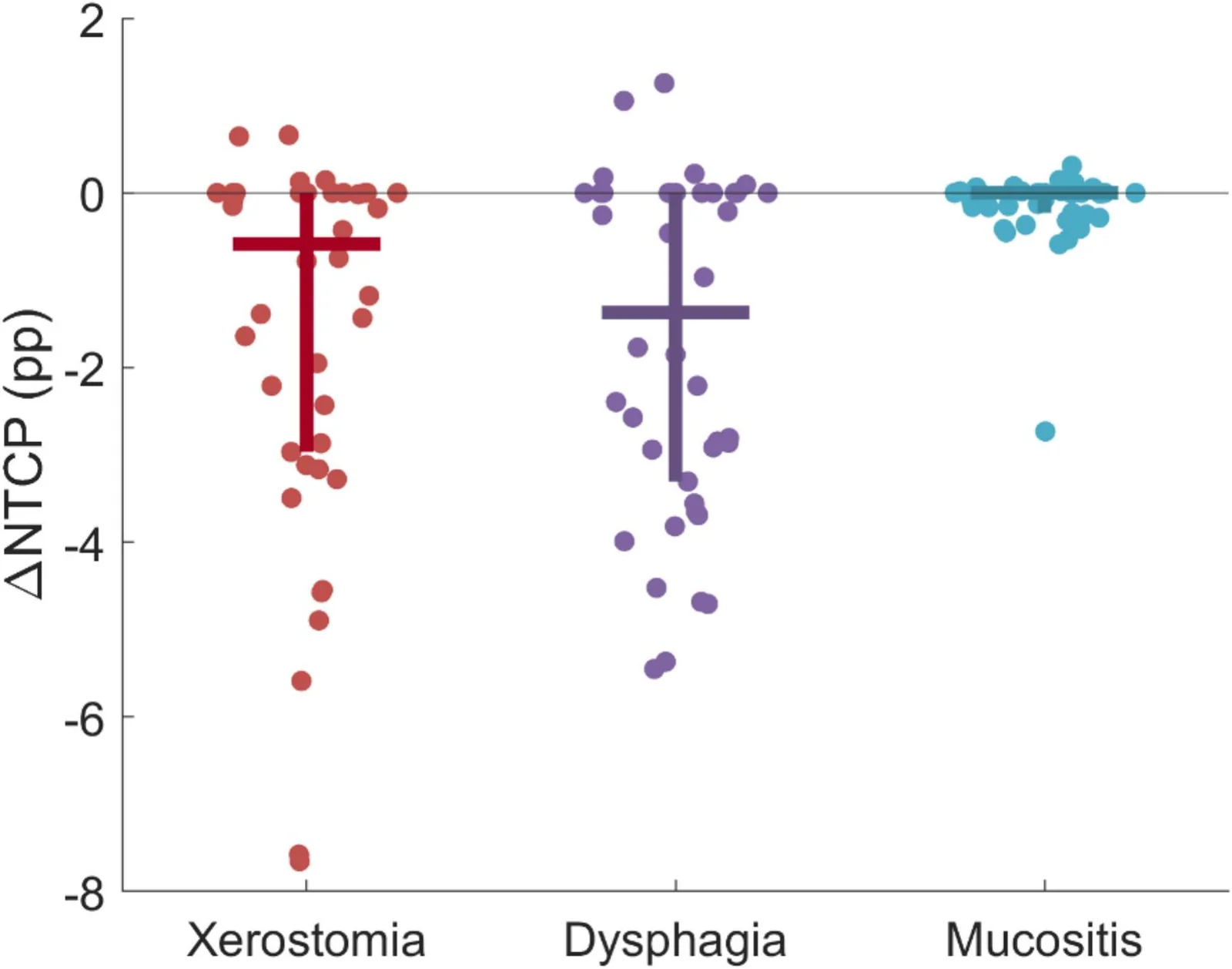
Difference in NTCP between the adapted plans and the original plans in the complete cohort, in percentage points (pp).

## 4 Discussion

This study demonstrates that adapting PORT plans based on SLNB for OTSCC can potentially enhance treatment safety and significantly reduce expected toxicity. We found that nearly 50% of patients exhibited individual lymphatic drainage patterns not covered by our standard treatment protocol, suggesting a substantial risk of omitting clinically relevant neck levels harboring metastasis. Clinical relevance is nonetheless uncertain because if the SN is negative, it might be safe to omit a level regardless. By preparing adapted RT plans, and estimating the corresponding NTCP, we showed that omitting contralateral neck in the absence of contralateral drainage would lead to a significant reduction in predicted toxicity. As expected, the risk of acute mucositis (G3+) was not very different between the plans, and the absolute reduction in the risk of late xerostomia (G3+) and dysphagia (G2+) may appear modest at around 3 pp. However, these are considered severe side effects, with a notable impact on patients' quality of life, and the absolute risk of such side effects was already low with the standard treatment plans. Therefore, the estimated reduction in the reduced risk group is clinically meaningful.

In OTSCC, Novikov et al. [20]. explored the use of SPECT imaging to characterize lymphatic drainage patterns and assess the potential impact on radiotherapy planning. Their cohort included 26 patients with early-stage OTSCC, in whom bilateral lymphatic drainage was detected in 38.5%, a proportion higher than observed in our study. Three patients received PORT, and reconstructed treatment plans omitting the contralateral neck demonstrated significant dose reductions to the spinal cord and contralateral parotid gland, supporting the feasibility of lymph flow–guided de-escalation.

Beyond oral tongue–specific cohorts, a prospective feasibility study evaluating a SPECT/CT-based SN procedure to guide unilateral elective nodal irradiation in lateralized head and neck squamous cell carcinoma planned for primary radiotherapy further demonstrated the feasibility of this approach [21]. In this study, SPECT/CT successfully identified SNs and lymphatic drainage patterns suitable for treatment adaptation, with frequent visualization of unilateral drainage pathways and SNs located outside conventional elective nodal volumes. These findings reinforced the concept that anatomy-based elective nodal irradiation may overtreat a substantial proportion of patients, and that SPECT/CT mapping can provide actionable information to safely individualize target volumes.

The SUSPECT trial explored SPECT-guided radiotherapy in the primary treatment setting for a mixed cohort of head and neck cancers [22]. Although oral cavity cancers were included, the number of OTSCC patients was not specified. The study enrolled patients with lateralized T1–3N0–2bM0 disease, of whom 50 received SPECT-guided elective nodal irradiation, with outcomes compared to historical controls. Only one contralateral regional recurrence was observed. Importantly, SPECT-guided radiotherapy was associated with significantly reduced rates of dysphagia, feeding-tube dependency, and xerostomia, highlighting its potential to reduce treatment-related morbidity.

These results led to the development of SUSPECT-2, a successor study designed to further validate this strategy by incorporating contralateral sentinel node procedures when contralateral drainage is visualized; however, only the study protocol has been published to date [23]. In parallel, the PRIMO randomized controlled trial is ongoing and investigates sentinel lymph node biopsy–guided radiotherapy in the primary setting. Notably, PRIMO includes oropharyngeal, hypopharyngeal, and laryngeal cancers, but excludes oral cavity tumors, and does not exclude lesions close to the midline [24].

Finally, a phase I trial by Longton et al. evaluated SPECT-modified radiotherapy in 16 clinically N0 head and neck cancer patients treated in the primary setting with the purpose of reducing elective nodal volumes and demonstrated excellent regional control, along with reduced median NTCP for xerostomia, dysphagia, and hypothyroidism [25].

In summary, across multiple early-phase and proof-of-concept studies, SPECT- and SLNB–guided approaches consistently demonstrate the feasibility of individualizing elective neck irradiation in head and neck cancer. These studies show that lymphatic drainage patterns are heterogeneous, even in lateralized and early-stage diseases, and that tailoring nodal target volumes based on individual lymph flow can maintain regional control while substantially reducing radiation exposure to OAR. Although data specific to OTSCC remain limited and largely non-randomized, the available evidence suggests a meaningful potential to reduce treatment-related toxicity without compromising oncologic safety. Ongoing and future prospective trials will be essential to confirm these findings and to define the role of lymphatic pathway–guided radiotherapy in routine clinical practice.

A limitation of our study is its retrospective nature which makes it difficult to directly translate the clinical usefulness of SN based treatment planning to routine clinical practice. Although SPECT/CT and SLNB provide individualized information on lymphatic drainage, limitations in tracer detection and spatial resolution may, although rarely, result in clinically relevant drainage pathways remaining unrecognized. Tumor-related lymphatic obstruction could also potentially alter normal drainage and redirect lymphatic flow through alternative pathways. For this reason, SN-based treatment de-escalation would primarily be applicable to patients with early-stage T1–T2 tumors. Furthermore, we only analyzed OTSCC patients so we cannot conclude if the results apply to other head and neck cancer sites where drainage patterns can be different and we acknowledge the limited number of patients in this study that effects generalizability and precision. One of the strengths of this study is using NTCP models to predict reduction in toxicity. The NTCP models were not validated for our cohort, but the models for xerostomia and dysphagia were developed on relatively large patient materials which we believe makes them clinically relevant for OTSCC patients.

The models were applied to both A) the target volumes used for the actual treatment, and B) to the synthetic target volumes of the current study. While both are expected to fall within the range of a general head and neck cancer cohort, making them suitable for evaluation with the selected models, the estimates are associated with uncertainties. However, the estimated differences in NTCP between the plans are less sensitive to these uncertainties, compared to the absolute values.

## 5 Conclusion

In conclusion, modifying adjuvant radiotherapy planning based on SLNB results for patients with OTSCC could lead to more individualized treatment and may increase safety and reduce side effects. We are currently planning to validate this with a prospective study with appropriate safety monitoring.

## CRediT authorship contribution statement

**Einar Björgvinsson:** Writing – review & editing, Writing – original draft, Visualization, Data curation, Conceptualization. **Aeneas Kolev:** Writing – review & editing, Methodology, Investigation, Data curation, Conceptualization. **Eva Onjukka:** Writing – review & editing, Visualization, Software, Methodology, Formal analysis. **Mattias Hedman:** Writing – review & editing, Supervision, Resources. **Åsa Carlsson Tedgren:** Writing – review & editing, Supervision, Resources, Funding acquisition. **Lars Södergren:** Software, Investigation. **Signe Friesland:** Writing – review & editing, Supervision, Resources, Conceptualization. **Gregori Margolin:** Writing – review & editing, Investigation, Data curation, Conceptualization. **Lars-Olaf Cardell:** Supervision, Funding acquisition, Conceptualization. **Michael Gubanski:** Writing – review & editing, Supervision, Methodology, Investigation, Formal analysis, Data curation, Conceptualization.

## Ethics declaration

### Informed consent and patient details

Written informed consent to take part in the study and to publish the article has been obtained from all participants or their legal representatives. The privacy rights of participants have been observed.

### Studies in human

This study was performed in compliance with relevant laws, regulatory frameworks and guidelines where the research took place. This study was approved by the The regional ethics committee in Stockholm. (Approval No. Protocol numbers 2013/1943–31/4, 2019–03518 and 2024–01475-02).

## Declaration of competing interest

The authors declare that they have no known competing financial interests or personal relationships that could have appeared to influence the work reported in this paper.

## Figures and Tables

**Table:** 

OAR/PRV⁎	Restrictions	EQ.D2#	34 fractions (Gy)	25 fractions (Gy)	α/β
Spinal canal	D0.1cc (Gy)	43.4	50	45.5	2
PRV spinal canal	D0.1cc (Gy	48.4	54	42.9	2
Brain stem	D0.1cc (Gy)	56.5	60	54.2	2
Brain stem interior	D0.1cc (Gy)	48.4	54	49	2
Parotid glands bilateral	Mean dose (Gy)	18.7	25	23.7	3
Parotid gland contralateral	Mean dose (Gy)	14.4	20	19.1	3
Submandibular glands	Mean dose (Gy)	32.3	39	36.3	3
Oral cavity	Mean dose (Gy)	23.3	30	28.2	3
Larynx	Mean dose (Gy)	33.4	40	37.2	3
Pharyngeal constrictor	Mean dose (Gy)	44.7	50	46.1	3
Mandible and temporo mandibular joint	D1cc (Gy)	68	68	62	3

Color: Yellow- Prioritized over target coverage; Green- High priority; Blue- If possible.

⁎ PRV- Planning organ at risk volume, #Equivalent dose in 2 Gy fractions.

## Appendix A

Dose planning: optimization constraints and prioritizing
